# Complications of hepatic echinococcosis: multimodality imaging approach

**DOI:** 10.1186/s13244-019-0805-8

**Published:** 2019-12-02

**Authors:** Silvia Greco, Roberto Cannella, Dario Giambelluca, Giusy Pecoraro, Emanuele Battaglia, Massimo Midiri, Giuseppe Brancatelli, Federica Vernuccio

**Affiliations:** 1 Dipartimento di Biomedicina, Neuroscienze e Diagnostica avanzata (BIND), University of Palermo, Palermo, Italy; 2 Department of General Surgery and Emergency, University of Palermo, Via del Vespro 127, 90127 Palermo, Italy; 3 Department PROMISE (Department of Health Promotion, Mother and Child Care, Internal Medicine and Medical Specialties), University Hospital of Palermo, Piazza delle Cliniche, 2, 90127 Palermo, Italy; 4 University Paris Diderot, Sorbonne Paris Cité, Paris, France; 5 I.R.C.C.S. Centro Neurolesi Bonino Pulejo, Contrada Casazza, SS113, 98124 Messina, Italy

**Keywords:** Echinococcosis, Multimodal imaging, Computed tomography, Endoscopic retrograde cholangiopancreatography

## Abstract

Hydatid disease is a worldwide zoonosis endemic in many countries. Liver echinococcosis accounts for 60–75% of cases and may be responsible for a wide spectrum of complications in about one third of patients. Some of these complications are potentially life-threatening and require prompt diagnosis and urgent intervention. In this article, we present our experience with common and uncommon complications of hepatic hydatid cysts which include rupture, bacterial superinfection, and mass effect-related complications. Specifically, the aim of this review is to provide key imaging features and diagnostic clues to guide the imaging diagnosis using a multimodality imaging approach, including ultrasound (US), computed tomography (CT), magnetic resonance (MR), and endoscopic retrograde cholangiopancreatography (ERCP).

## Key points

- Rupture of hepatic hydatid cyst occurs in about 35% of cases: “waterlily sign,” “snake/serpent sign,” or the “snowstorm pattern” suggest contained rupture of hepatic hydatid cyst; intracystic fat suggests communicating rupture of hepatic hydatid cyst within the biliary tree.
- Superinfection of hepatic hydatid cyst may lead to higher incidence of postoperative complications and life-threatening complications.
- Hepatic hydatid cysts may grow in size and lead to significant mass effect on adjacent structures, including portal vein, hepatic veins, biliary tree, right diaphragm, stomach, and kidney.

## Background

Hydatid disease—also known as echinococcosis—is a worldwide zoonosis caused by the larvae of *Echinococcus* tapeworm [1]. It is estimated by the World Health Organization (WHO) that more than 1 million people are affected with hydatid disease [2], with incidence rates in humans that can exceed 50 per 100,000 person-years in endemic regions and associated annual costs of US$ 3 billion [1]. The two main forms of the disease in humans include cystic echinococcosis—due to *E. granulosis*—and, less commonly, alveolar echinococcosis—due to *E. multilocularis* [3]. Echinococcosis due to *E. granulosis* mainly affects the liver and lungs and less frequently the bones, kidneys, spleen, muscles, and central nervous system [2, 3].

Liver echinococcosis accounts for 60–75% of cases, with 80% of hydatic cysts located in the right hepatic lobe [3, 4]. Liver echinococcosis is usually asymptomatic and may have a wide variety of imaging presentations at diagnosis—e.g., simple uniloculated cysts, cysts with intralesional daughter cysts, coarse wall, or intralesional calcifications—mainly depending on the stage of the disease [3–6]. The course of liver echinococcosis may be associated with a wide spectrum of complications in about one third of patients [7]. Some of these complications are potentially life-threatening and, therefore, require prompt diagnosis and urgent intervention [3, 7]. In this article, we present our experience with common and uncommon complications of hepatic hydatid cysts which include rupture, bacterial infection, and mass effect-related complications. Specifically, the aim of this review is to provide key imaging features and diagnostic clues to guide the imaging diagnosis using a multimodality imaging approach, including US, CT, MR, and endoscopic retrograde cholangiopancreatography (ERCP).

### Complications of hepatic hydatid disease: rupture of hepatic hydatid cysts

Rupture of hepatic hydatid cysts occurs in about 35% of cases, with communicating rupture being the most common type (15%), followed by contained rupture (12%) and direct rupture (6%) [7]. Hepatic hydatid cyst rupture is mainly caused by degeneration of hydatid membranes. Communicating rupture refers to the passage of hydatid material from the hepatic hydatid cyst into the biliary tract, peritoneal cavity, or other adjacent organs. This complication is favored by prior surgery or percutaneous treatment for echinococcosis, leading to an incidence of up to 50% in these patients [8].

#### Contained rupture of hepatic hydatid cysts

**Fig. 1:**
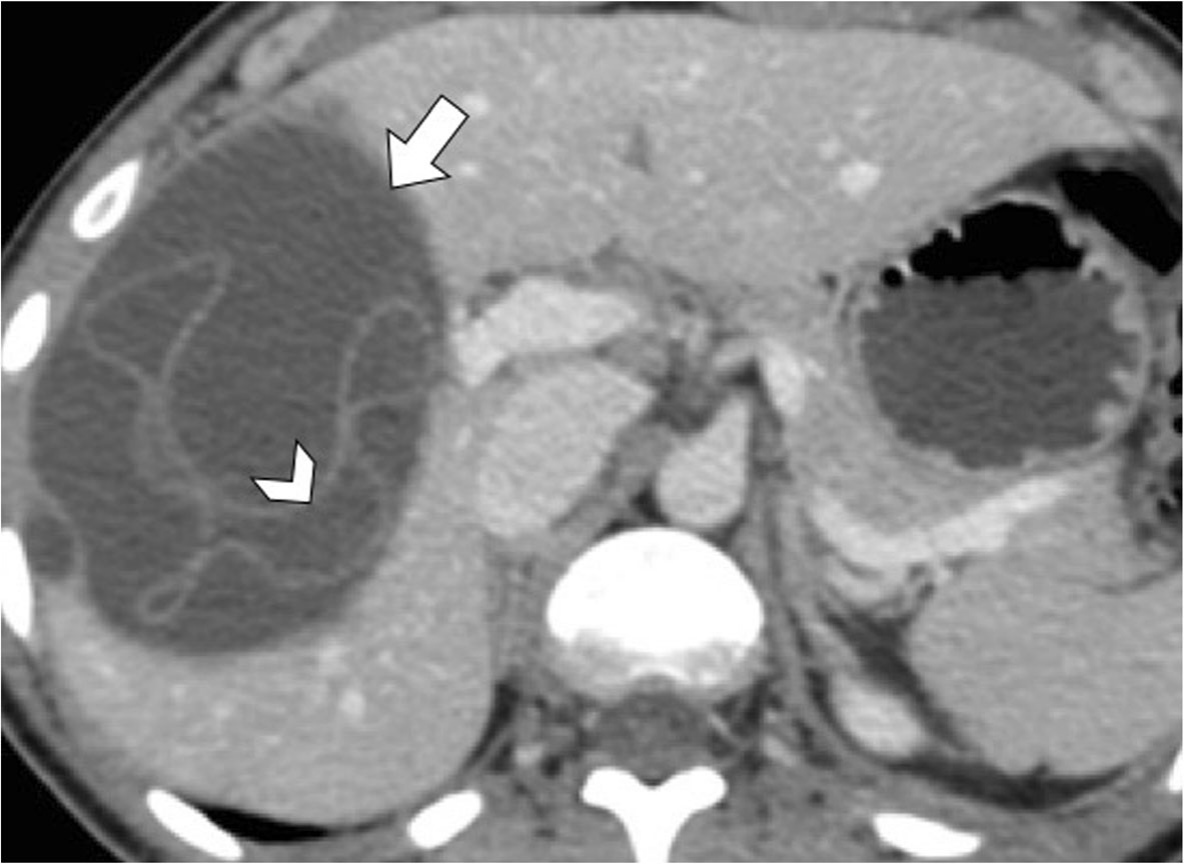
A 50-year-old woman with contained rupture hydatid cyst. Axial contrast-enhanced CT on portal venous phase shows hepatic hydatid cyst (arrow) with hyperattenuating serpentine linear membrane within the cyst (arrowhead)

**Fig. 2:**
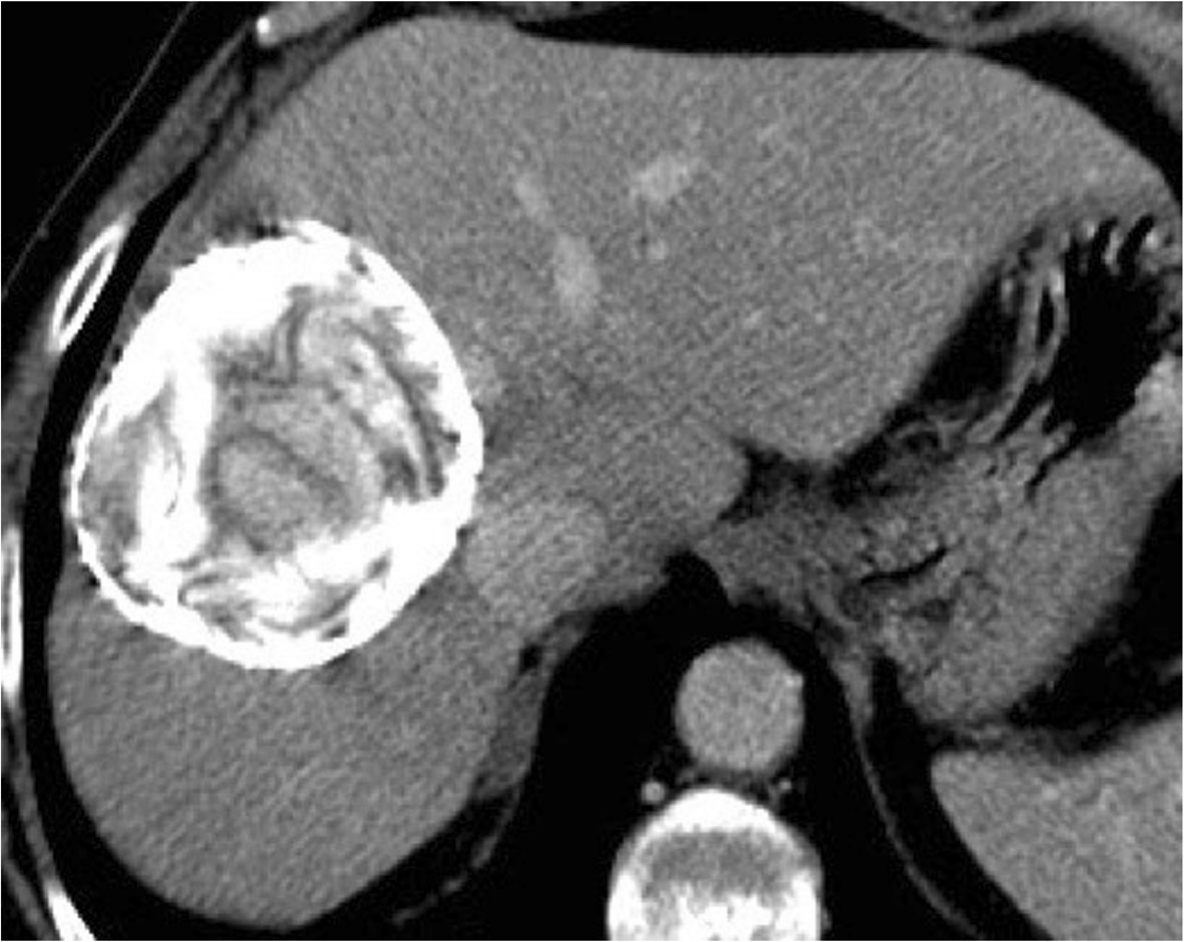
A 65-year-old woman with contained rupture hydatid cyst due to response to therapy. Contrast-enhanced CT on portal venous phase shows the serpentine linear membrane within an almost completely calcified cyst

Contained rupture occurs when the endocyst ruptures within the cyst while the outer pericyst remains intact. The causative mechanism is an augmented intracystic pressure due to hydatid fluid collection in the space between the pericyst and the endocyst as a result of hepatic hydatid cyst degeneration, trauma, or response to therapy [5]. Typical imaging signs suggesting the diagnosis of contained rupture are “waterlily sign” (i.e., free-floating membranes within the cyst), “snake/serpent sign” (i.e., discrete, curvilinear structures found within a complex cyst), or the “snowstorm pattern” (i.e., swirling of hydatid sand within hydatid cysts) due to detached undulating membranes within the cyst that appear on ultrasound, CT, and MR as serpentine floating linear membranes within the cyst (Fig. 1) [7, 9]. When contained rupture occurs after therapy response or natural degeneration, the detached membranes may show internal hyperattenuation or calcifications (Fig. 2) [10].

#### Communicating rupture of hepatic hydatid cysts within the biliary tree

Communicating rupture within the biliary tree is more common in large cysts (i.e., up to 80% of hepatic hydatid cysts larger than 7.5 cm), located in the central liver segments, and in advanced stages of the disease [11]. The causative mechanism is an increased intracystic pressure, which leads to compression and necrosis of the wall of adjacent bile ducts, followed by incorporation of small biliary ducts within the pericyst and then rupture of the biliary ducts [8, 11]. Communicating rupture within the biliary tree may present as small fissures that are usually asymptomatic, or perforation (i.e., frank communication), that may clinically cause obstructive jaundice and cholangitis [7]. In both cases, hydatid sand or daughter cysts may be cleared into the biliary tract and may be responsible for acute pancreatitis [11].

**Fig. 3:**
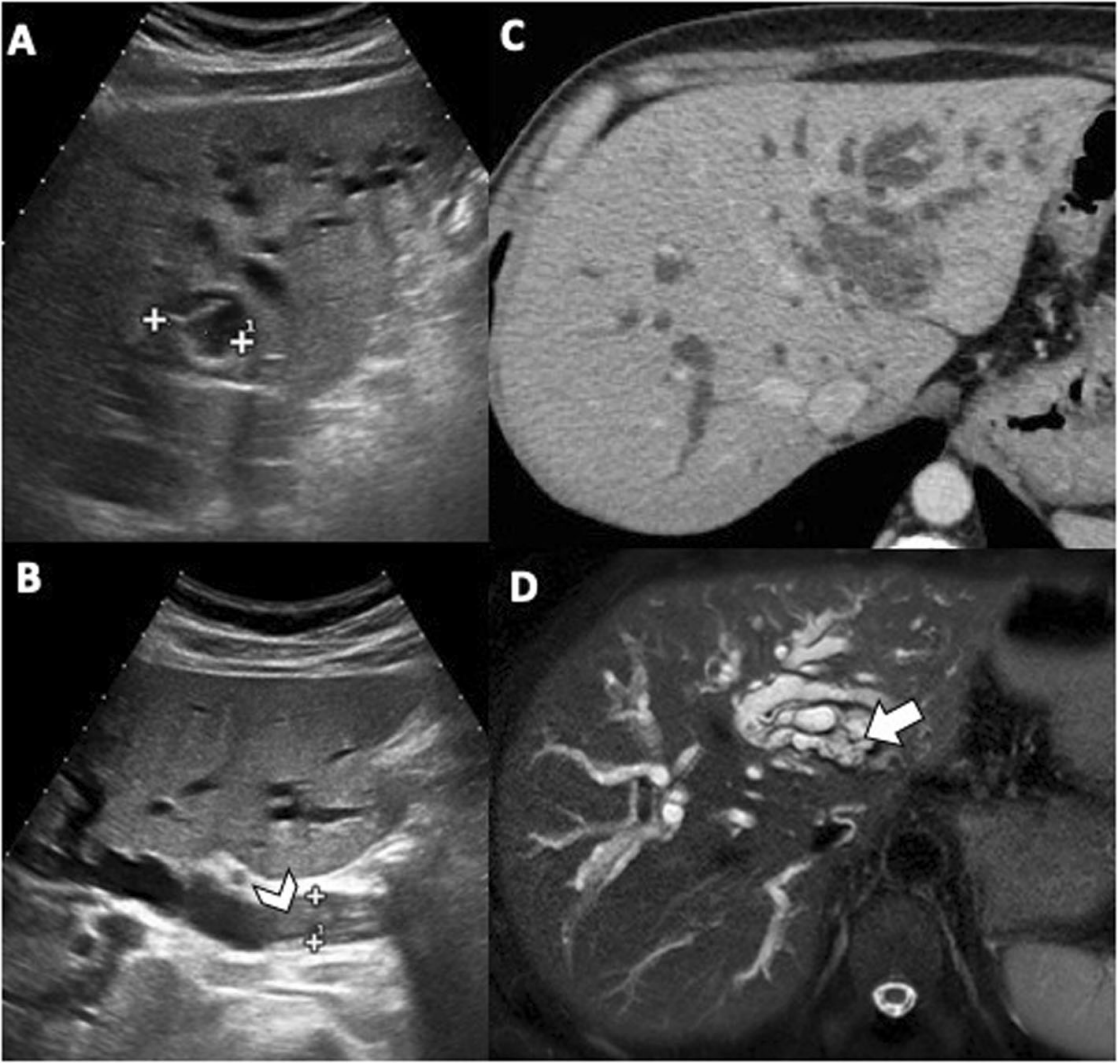
A 48-year-old man with hydatid liver cyst complicated with communicating rupture. **a**, **b** US images show a focal anechoic liver lesion with internal septa and hyperechoic peripheral rim, with some posterior shadows, diffuse biliary dilatation and endoluminal hyperechoic foci (arrowhead) into the common bile duct with no posterior acoustic shadows. Axial CT scan in the portal venous phase (**c**) and MR image on T2-weighted sequence (**d**) confirm the presence of a liver cyst and diffuse biliary dilatation, with material into the biliary tree (arrow). These features are consistent with progressive migration of the hydatid sand and membranes from the hilar confluence to the common bile duct

**Fig. 4:**
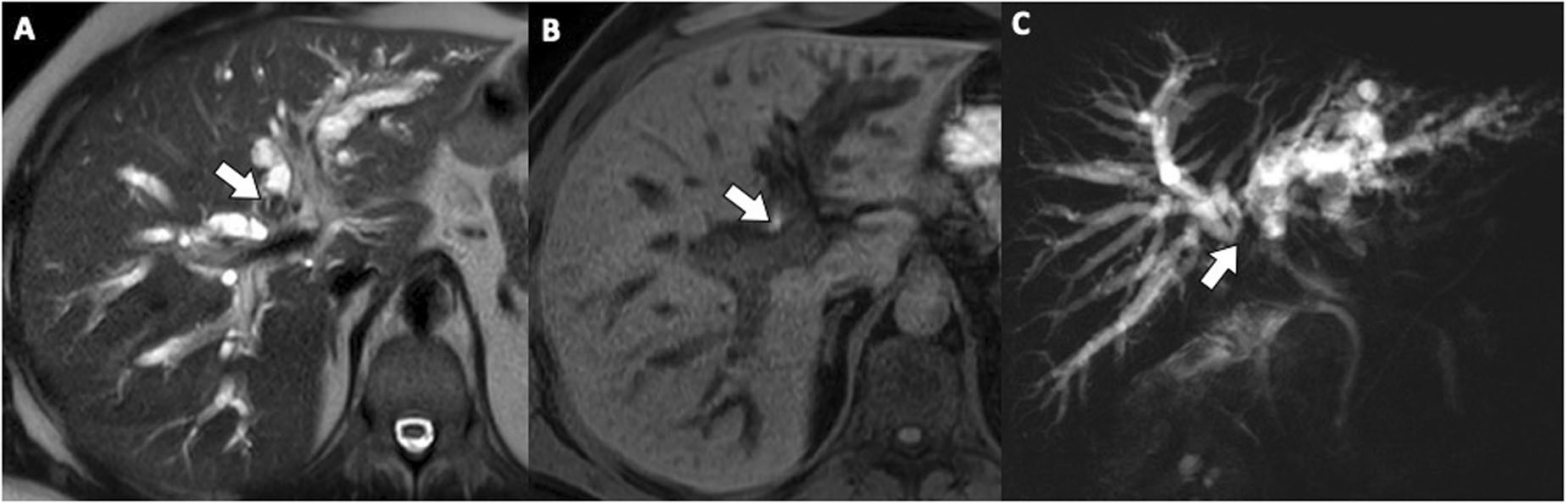
A 48-year-old man with communicating rupture. MR images on T2-weighted (**a**) and T1-weighted gradient echo (**b**) sequences demonstrate endoluminal biliary content (arrows), hypointense and hyperintense on respective images, at the hilar biliary confluence. On MRCP image (**c**), the endoluminal obstructive biliary content leads to loss of signal intensity (arrow) from the hilar biliary confluence to the common hepatic duct, while the common bile duct is patent

**Fig. 5:**
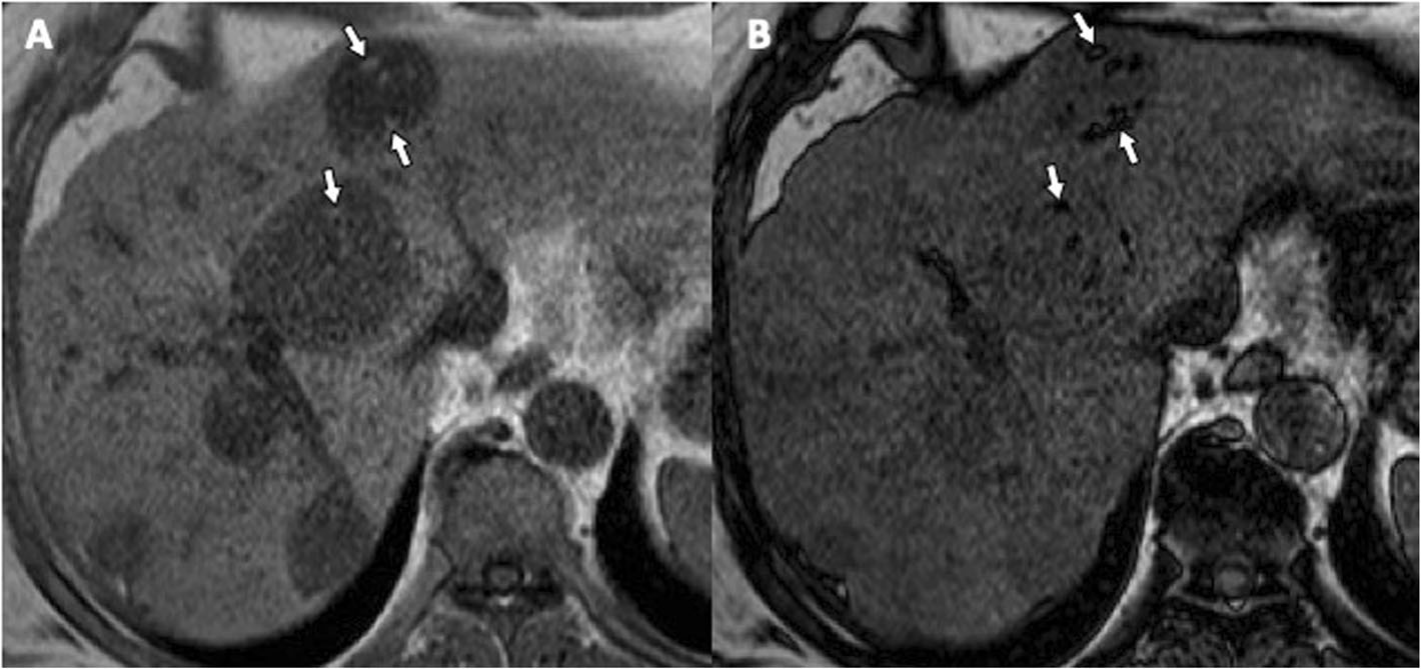
A 66-year-old man with multiple fat-containing hydatid cysts in the liver. **a** Axial gradient-echo in-phase T1-weighted MR image shows multiple high signal intensity foci (arrows) within hepatic hydatid cyst. **b** Axial gradient-echo opposed-phase T1-weighted MR image shows macroscopic fat within the same lesions, a finding confirmed by chemical shift artifact and signal cancelation (arrows) surrounding the lipid pure component

**Fig. 6:**
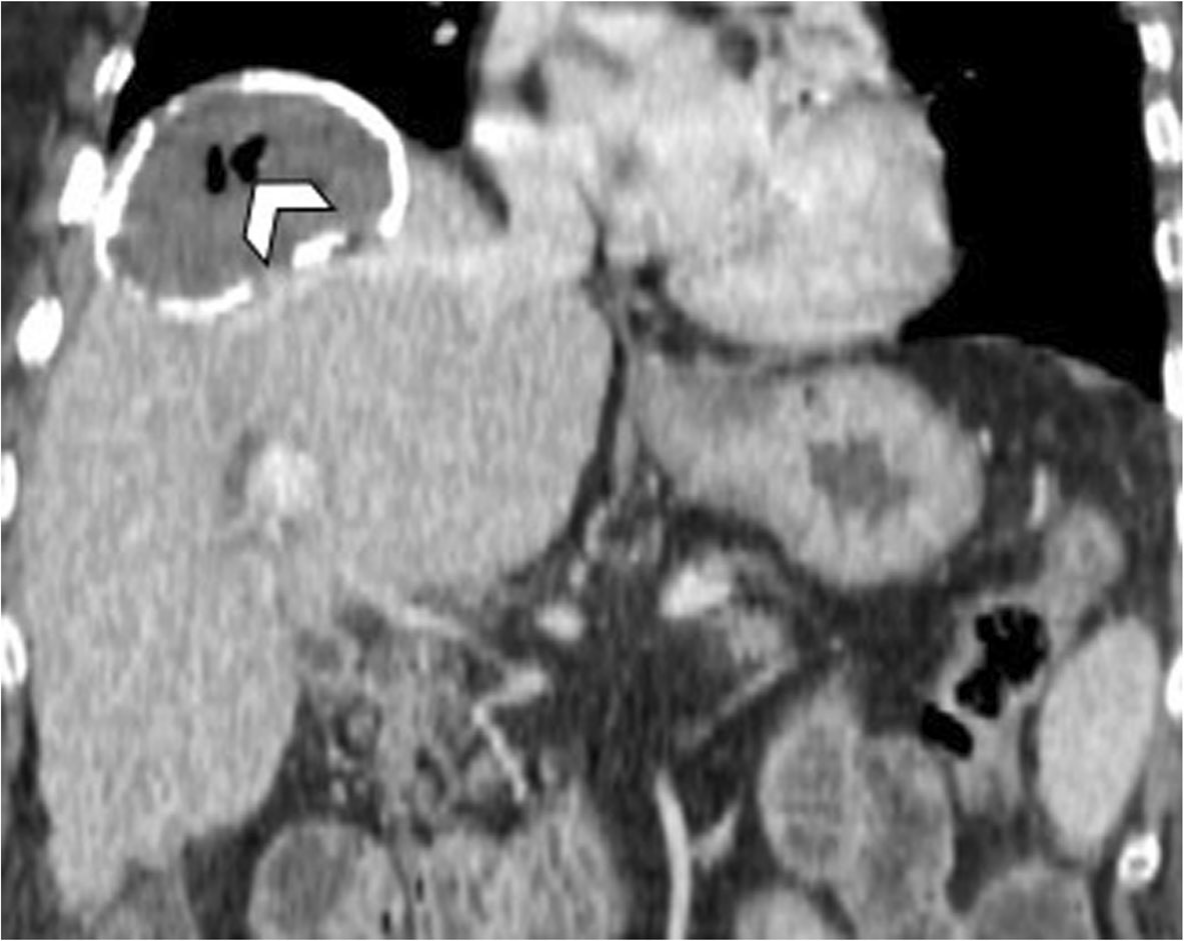
A 54-year-old man with air content within a hepatic hydatid cyst. Coronal reformatted CT image on portal venous phase shows air bubbles (arrowhead) within a unilocular calcified hydatid cyst. This finding was suggestive of occult cysto-biliary communication, which was later confirmed at surgery

**Fig. 7:**
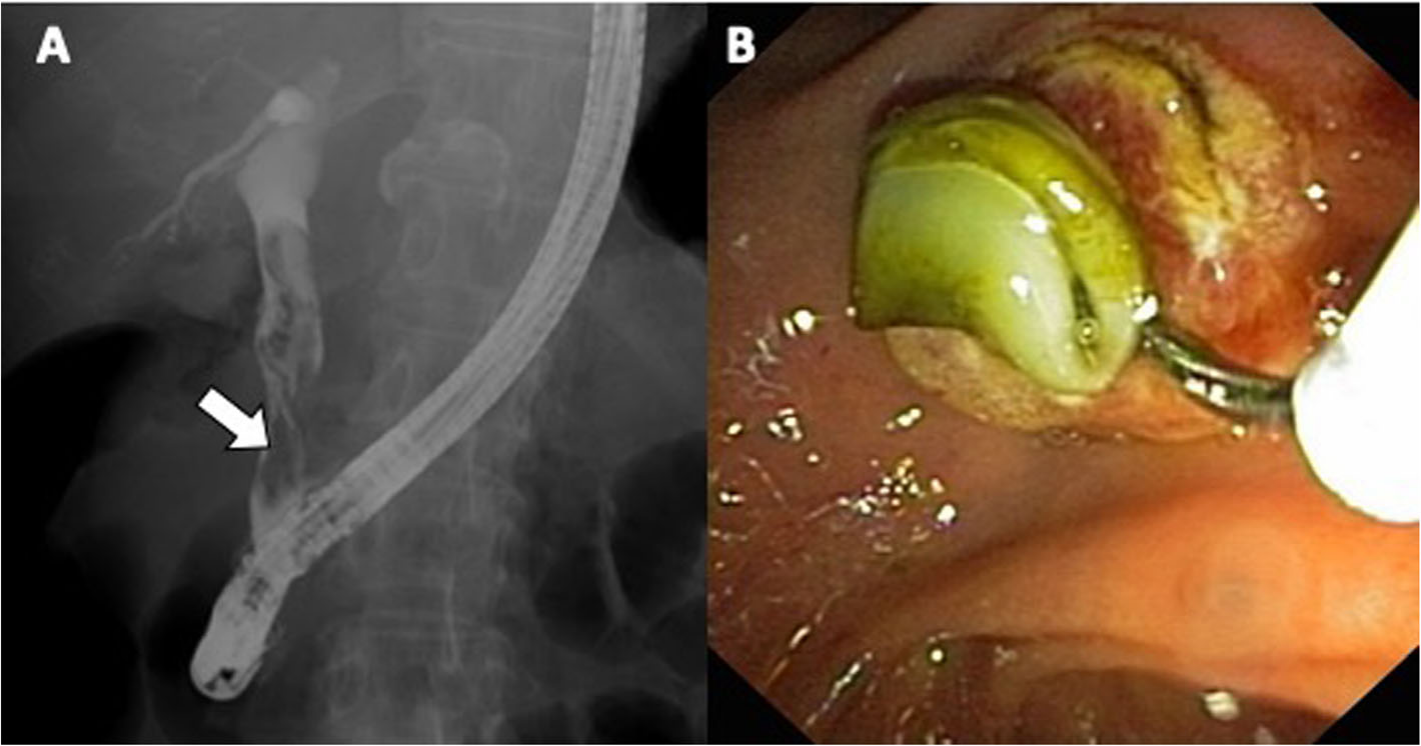
A 65-year-old woman with cysto-biliary rupture. **a** Radiographic image performed during ERCP procedure shows multiple filling defects into the common bile duct (arrow), representing hydatid membranes. **b** Endoscopic image of the same patient shows hydatid membranes extraction

**Fig. 8:**
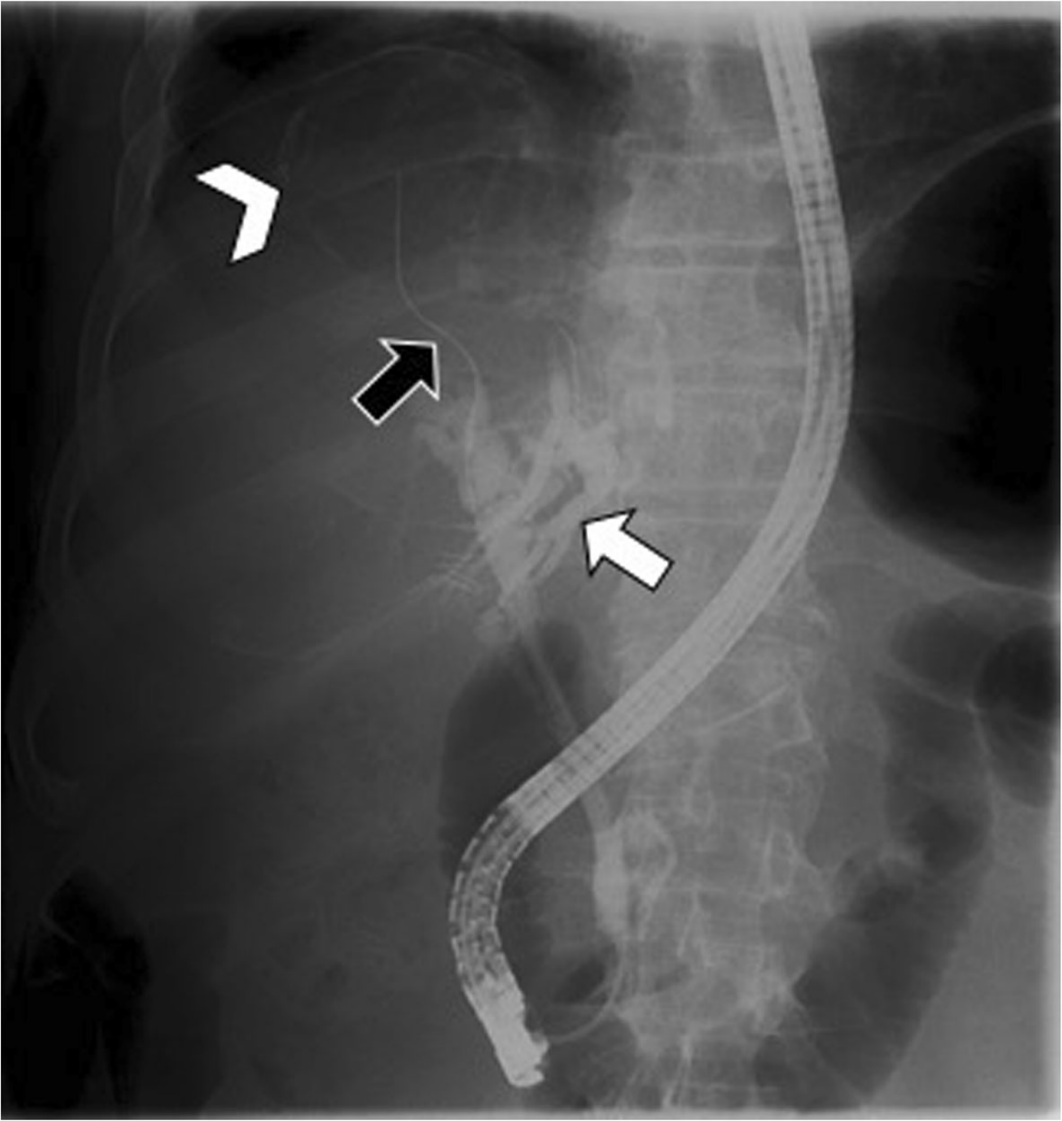
A 72-year-old man with communicating rupture. Radiographic image performed during ERCP demonstrates a linear calcified opacity (arrowhead)—representing a hepatic hydatid cyst—and intrahepatic bile duct dilatation (white arrow). The catheter (black arrow) introduced in biliary tree enters directly into the cyst, documenting the cysto-biliary fistula

Communicating rupture of hepatic hydatid cysts within the biliary tree is usually seen on US as echogenic hepatic hydatid cyst with hypoechoic or snowstorm pattern; in case of perforation, rounded or linear hyperechoic structures without posterior shadowing can be detected within the common bile duct (Fig. 3) [7]. Hydatid material into the biliary tract is evident as high-attenuation/intensity content in the common bile duct on CT and T1-weighted MR imaging (Figs. 3 and 4) and as hypointense filling defect on T2-weighted MR imaging or magnetic resonance cholangiopancreatography (Figs. 3 and 4). MR images may show the direct fistulous track between the hepatic hydatid cyst and biliary tree as well as the leakage of hepatobiliary contrast agent from the biliary tract into the hepatic hydatid cyst when using hepatobiliary-specific contrast agent [12, 13]. Indirect signs of communicating rupture on CT and MR include the presence of intracystic fat (Fig. 5)—due to the lipid content of the bile—or air content and air-fluid levels (Fig. 6). ERCP has a high sensitivity (86–100%) in the diagnosis of intrabiliary rupture [14] as it directly demonstrates linear wavy filling defects of laminated hydatid membrane into the common bile duct (Fig. 7), the duodenum, or protruding from the ampulla of Vater, and may proof the communication with the introduction of a catheter from biliary ducts directly into the hepatic hydatid cyst (Fig. 8) [14]. However, ERCP may promote the formation of postoperative biliary fistulae [14], and therefore, MR should be preferred over ERCP for the diagnosis.

#### Communicating rupture of hepatic hydatid cysts into adjacent viscera

**Fig. 9:**
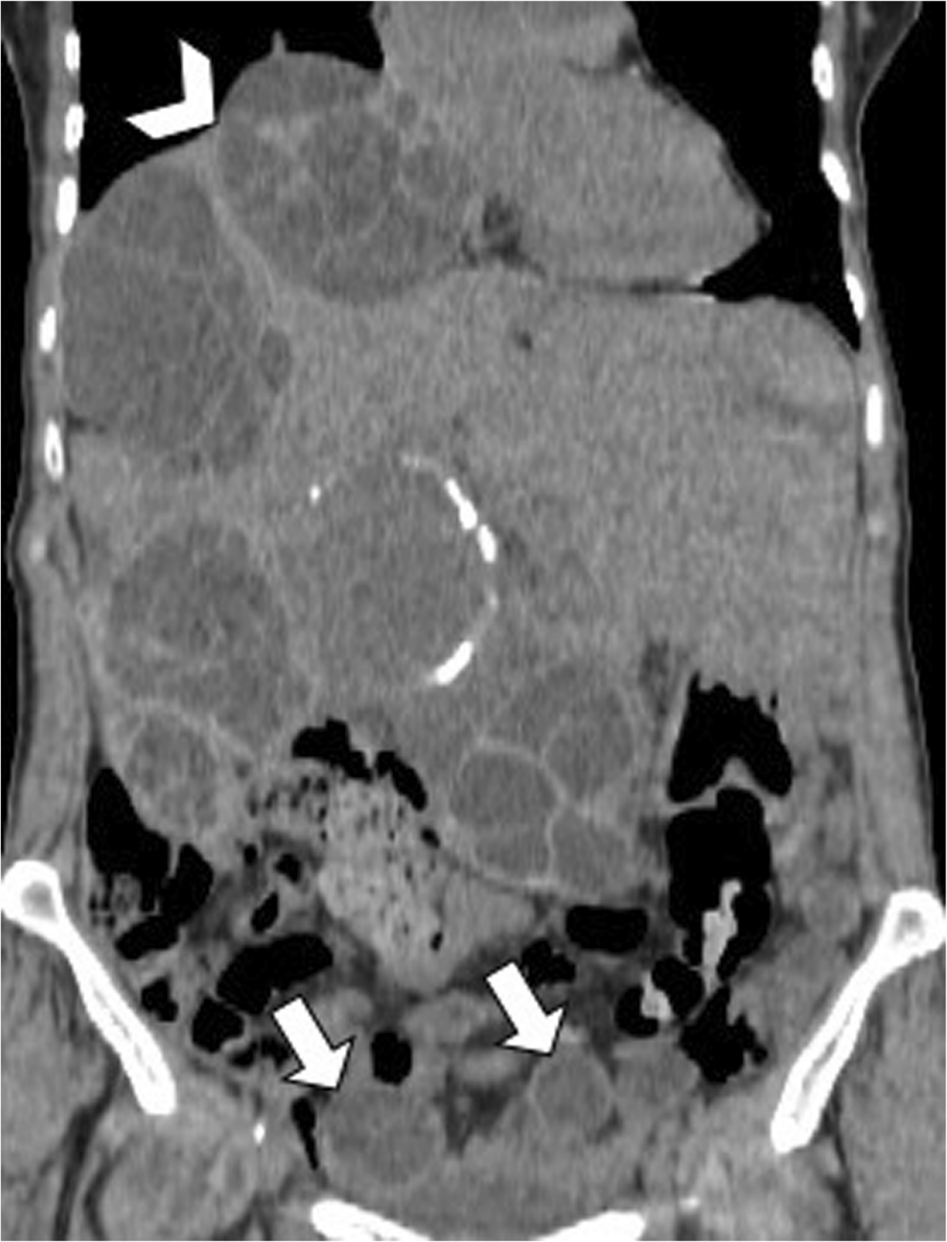
A 66-year-old woman with diffuse abdominal echinococcosis. Coronal reformatted CT image shows peritoneal dissemination of hydatid cysts (arrows), due to direct rupture of the original liver cysts and compression of the right diaphragm by one of the hepatic hydatid cyst (arrowhead)

**Fig. 10:**
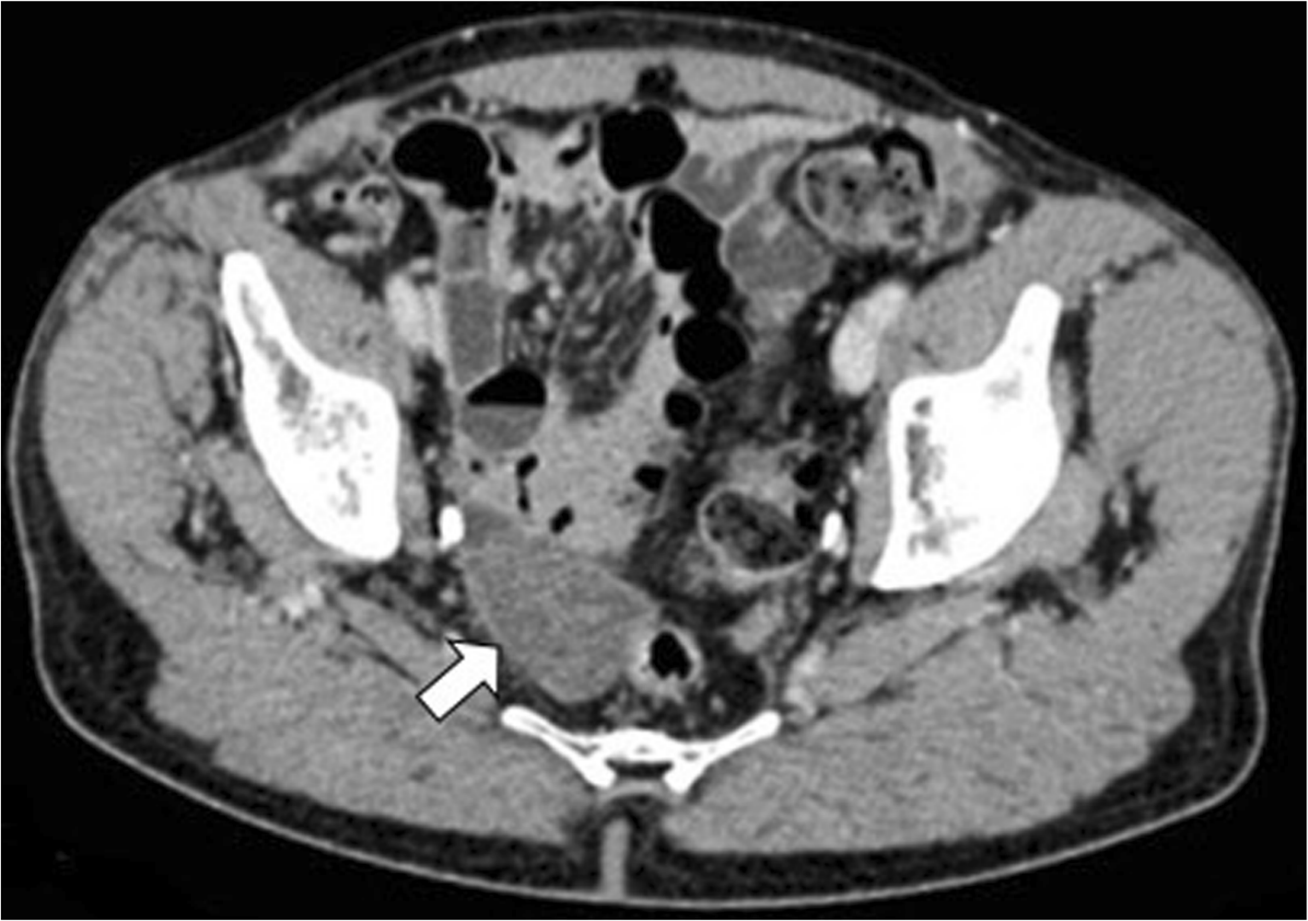
A 70-year-old man with echinococcosis. Axial CT image on portal venous phase demonstrates a pararectal homogeneous cystic lesion (arrow), showing internal septa and partial wall calcification, in a patient with hepatic hydatid cysts (not shown in this image)

**Fig. 11:**
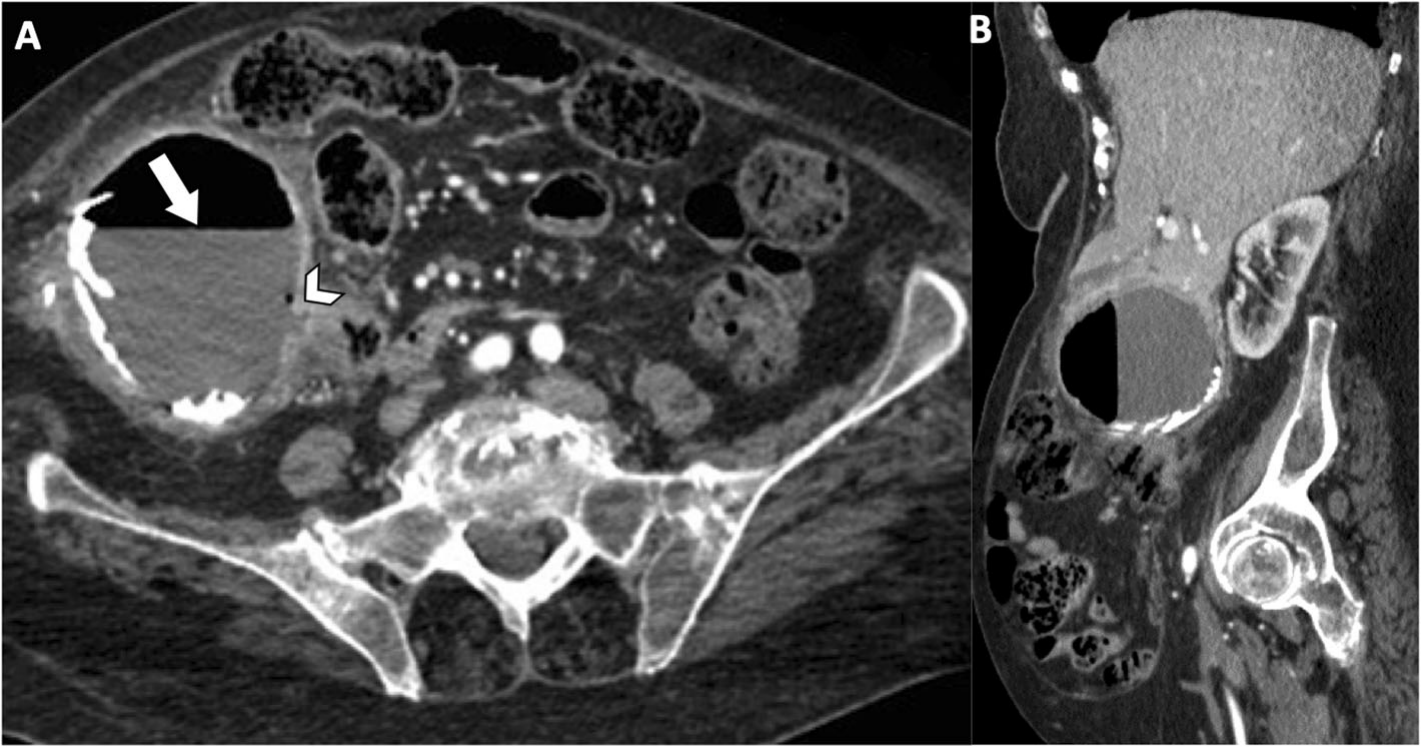
An 85-year-old woman with hydatid cysto-colic fistulization. Axial contrast-enhanced CT image (**a**) and sagittal reformatted CT image (**b**) on portal venous phase demonstrate a large hepatic hydatid cyst with peripheral wall calcifications and air-fluid level (arrow), with frank communication (arrowhead) with the right colon

**Fig. 12:**
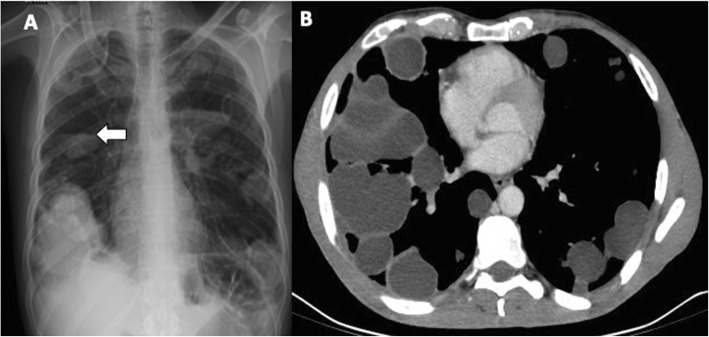
A 50-year-old man with hepatic and pleuro-pulmonary echinococcosis, secondary to trans-diaphragmatic dissemination. **a** Chest x-ray shows bilaterally multiple round cavitated lesions—some of which with air-fluid level (arrow)—as well as radiopaque lesions. **b** Chest CT scan of the same patient shows multiple cystic lesions, some of which appear stuffed with fluid-density material, others cavitated with air-fluid levels (not shown in this image)

Communicating rupture of hepatic hydatid cysts into adjacent viscera, peritoneal cavity, or pleural cavity is known as direct rupture and occurs in about 6% of cases [7]. Direct communication may be spontaneous or as a consequence of a minor or blunt abdominal trauma and results into leakage of hydatid material into the peritoneal or pleural cavity or in hollow viscera [15, 16]. Peritoneal dissemination may lead to implantation of hydatid scolices in several abdominal organs—i.e., *metastatic hydatidosis*—and may manifest dramatically as a life-threatening condition with anaphylactic shock or acute abdomen due to chemically induced peritonitis [15]. CT and MR imaging characteristically demonstrate daughter cysts within the peritoneal cavity (Figs. 9 and 10). Pelvic hydatid cysts may mimic adnexal cystic masses, and the identification of peripheral calcifications, floating membranes, or daughter cysts in hydatid cysts is crucial for differential diagnosis [17]. Communicating rupture of hepatic hydatid cysts with adjacent viscera—i.e., digestive tract (Fig. 11) or lungs (Fig. 12)—results into intracystic air-fluid levels related to the passage of air from bowel [18] or from the bronchial tree in case of bilioenteric or broncobiliary fistula, respectively [19]. The identification of parietal calcifications or direct passage of oral contrast from bowel into hepatic hydatid cyst are key imaging features for the diagnosis of communicating rupture of hepatic hydatid cyst with the digestive tract [18]. In patients with communicating rupture of hepatic hydatid cysts with the thoracic structures, chest x-ray and CT may show pleural effusion, elevation of the diaphragm, lung consolidation, or laminated atelectasis at the lung bases [19].

### Complications of hepatic hydatid disease: superinfection

**Fig. 13:**
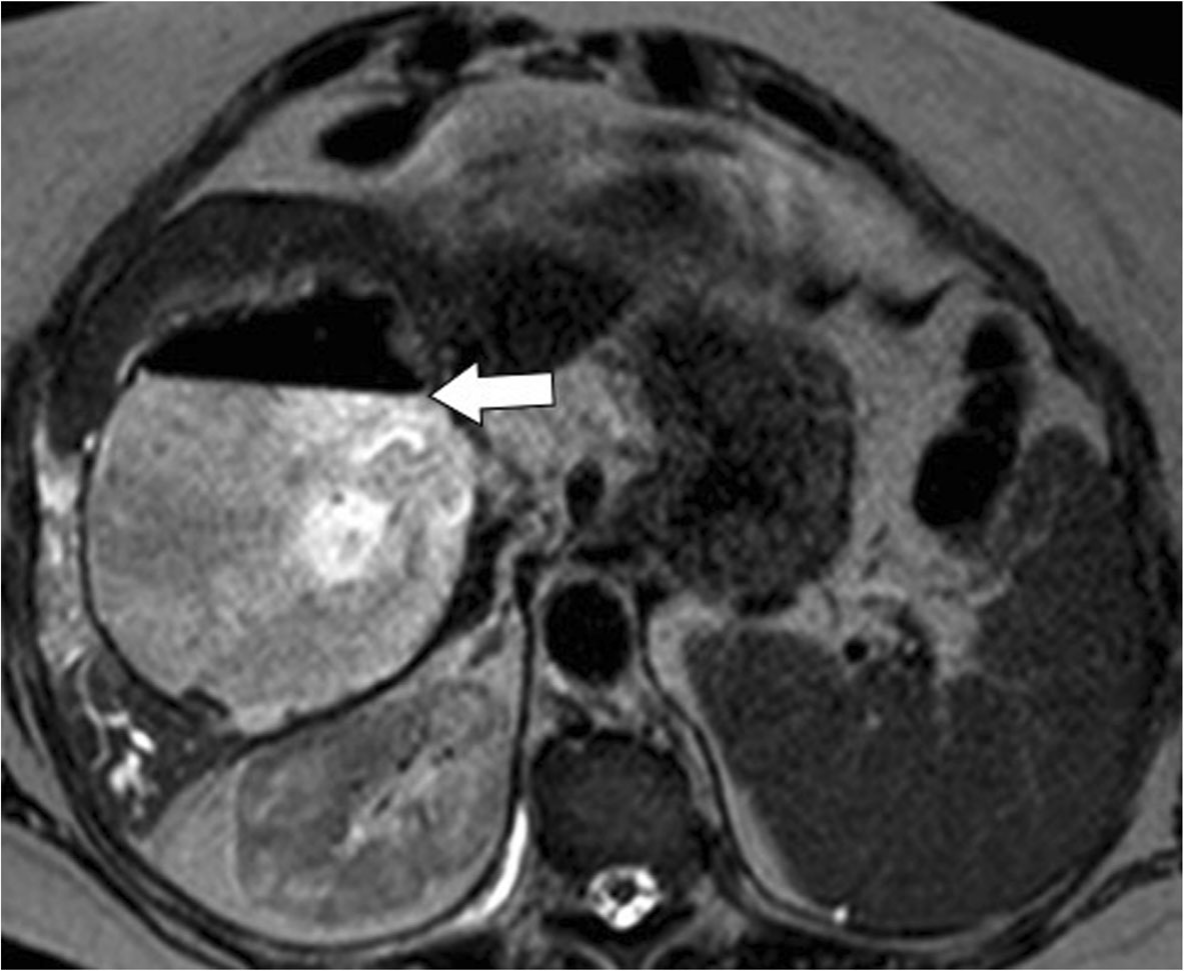
A 66-year-old woman with infected hydatid cyst. Axial T2-weighted MR image demonstrates an air-fluid level (arrow) within a calcified hydatid cyst, but lack of frank cysto-biliary communication. This finding suggests superinfection of a ruptured hydatid cyst that was later proved at surgery

**Fig. 14:**
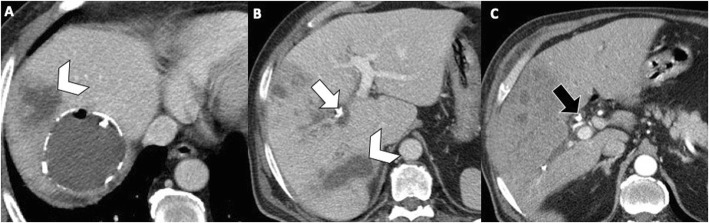
A 57-year-old man with communicating rupture and superinfection of liver hydatid cyst. Axial contrast-enhanced CT images at different levels (**a**, **b**) show multiple intrahepatic abscesses (arrowheads), adjacent to a hydatid cystic lesion with peripheral wall calcifications and intracystic air content. Note a calcified fragment of wall cyst within the right intrahepatic bile duct (white arrow), which migrates into the common bile duct (black arrow) at 10-days CT follow-up (**c**)

Superinfection may complicate ruptured hepatic hydatid cysts in up to 25% of cases [7, 20, 21]. Specifically, superinfection is more commonly associated with intrabiliary rupture and less frequently with peritoneal or intrabronchial rupture [21]. The disruption of the laminated membrane predisposes to superinfection and may lead to liver abscess, with fever and jaundice being the most common symptoms. Prompt recognition of superinfection is considered mandatory as it may lead to higher incidence of postoperative complications, life-threatening complications, and even death for septic shock [21–24]. US and CT may demonstrate multiple confluent intrahepatic lesions with poorly defined margins, associated with the presence of air and air-fluid level (Fig. 13) [4, 7]. However, these findings may also be present in uninfected ruptured cyst [4, 7]. Contrast-enhanced CT and MR may demonstrate an enhancing rim lesion in case of liver abscess and hypervascular areas in the contiguous liver parenchyma, which may reflect inflammatory changes occurring in case of superinfection (Fig. 14). The combination of these imaging findings with history of hepatic hydatid cyst, pain, fever, and leukocytosis should prompt the diagnosis of hepatic hydatid cyst suppuration.

### Complications of hepatic hydatid disease: mass effect-related complications

**Fig. 15:**
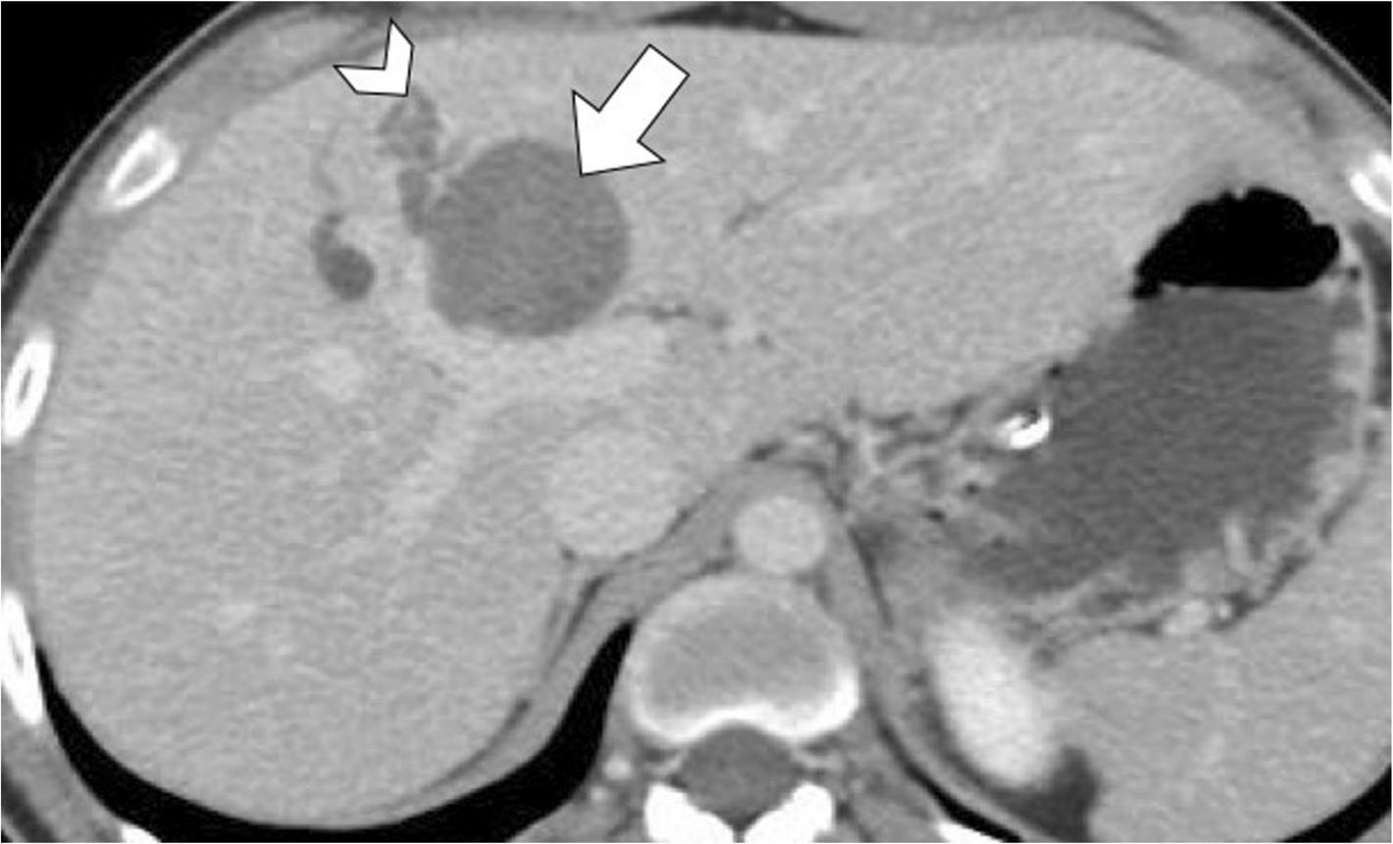
A 21-year-old man with hepatic hydatid cyst. Axial contrast-enhanced CT image on portal venous phase shows hepatic hydatid cyst (arrow) causing compression and dilatation of adjacent intrahepatic bile ducts (arrowhead)

**Fig. 16:**
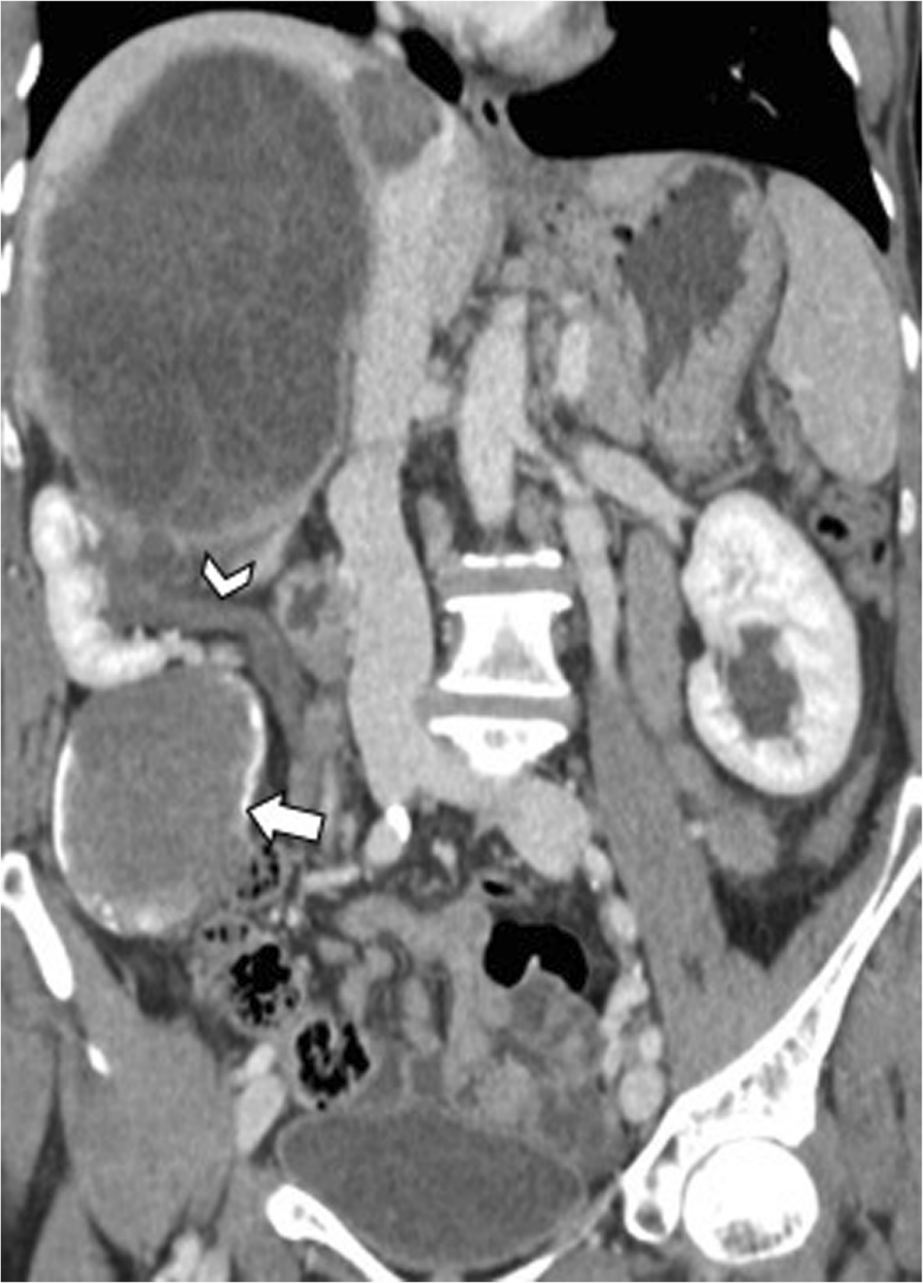
A 60-year-old woman with large partially exophytic hepatic hydatid cyst. Coronal reformatted CT image on portal venous phase shows a large hepatic hydatid cyst with multiple daughter cysts, associated with peritoneal hydatid cysts (arrow). The exophytic growth of the hepatic hydatid cyst causes significant mass effect on the right kidney, which appears compressed and dislocated with hydronephrosis (arrowhead)

**Fig. 17:**
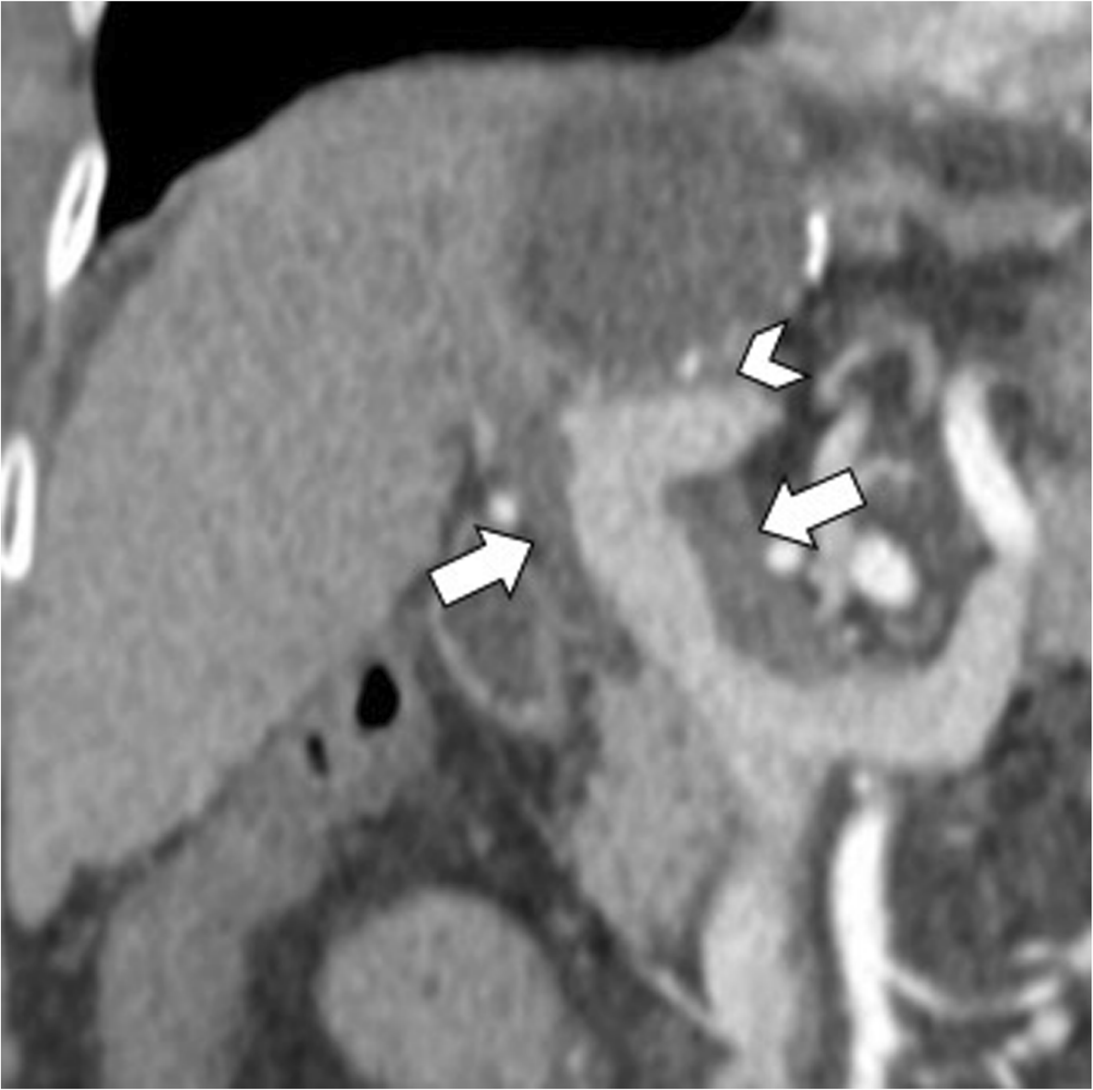
A 72-year-old woman with hydatid cyst and portal vein thrombosis. Oblique reformatted CT image on portal venous phase shows a large hepatic hydatid cyst adjacent to the left branch of portal vein (arrowhead) and partial portal vein thrombosis (arrows)

Hepatic hydatid cysts may gradually increase in size of about 1 cm in the first 6 months, and then of 2–3 cm each year depending on compressibility of surrounding structures [25]. Hepatic hydatid cysts growth may lead to significant mass effect on adjacent structures, including portal vein, hepatic veins, biliary tree (Fig. 15), right diaphragm (Fig. 9), stomach, and kidney (Fig. 16) [19, 26, 27]. Portal vein obstruction due to hepatic hydatid cyst may result into decreased portal vein inflow, thrombosis (Fig. 17), cavernous transformation of the portal vein, and hepatic morphological changes (i.e., atrophy of the involved lobe and compensatory hypertrophy of the contralateral lobe) [28]. However, portal hypertension in patients with hepatic hydatid cyst may also be caused by secondary sclerosing cholangitis or secondary biliary cirrhosis after surgery or use of parasiticides [29]. In patients with hepatic hydatid cyst and portal hypertension, possible complications include bacterial peritonitis, which may bring the patients to death [24]. Compression and displacement of hepatic veins and inferior vena cava may lead to secondary Budd-Chiari syndrome and development of collateral flow through azygous and hemiazygous veins [28]. Risk factors for Budd-Chiari syndrome secondary to hydatid cyst include large size of the cyst with involvement of two or more segments, posterior location of the cyst, previous surgery, and infection [30]. Clinical manifestations include abdominal pain, jaundice, and swelling of lower limbs [30]. In these patients, US and CT usually show hepatic hydatid cyst located in the hepatic dome, extrinsic compression of hepatic veins or inferior vena cava, and ascites [30]. Venous thrombosis may also develop in these patients [30]. Surgery is required for Budd-Chiari syndrome secondary to hepatic hydatid cyst; however, because of the risk of bleeding and possible co-existence of portal hypertension, a minimalist approach may be preferred in some cases [30].

### Diagnostic algorithm for diagnosis of hepatic echinococcosis

The diagnosis of hepatic hydatid cyst is based on epidemiology (i.e., patient living or coming from an endemic region), presence of pathognomonic features at imaging, and, in some cases, confirmation by various immunodiagnostic methods. Only in few cases histopathology or parasitology directly showing protoscolex or hooklets in cyst fluid is needed. Specifically, if US or CT imaging findings allow a confident diagnosis of hepatic hydatid cyst, no further test is usually needed, and patients can be treated depending on the stage of echinococcosis. Conversely, if reliable US or CT imaging diagnosis is not possible, MR imaging and further serological test can be performed to reach final diagnosis. In patients with history of hepatic echinococcosis, the onset of symptoms including fever, nausea and vomiting, jaundice, and raise of liver hepatic test should prompt the suspicion of complications. In regard to complications, US should be performed as first imaging technique, but it is usually not sufficient for the assessment of complications of hepatic hydatid cysts. Depending on availability and patient compliance, CT and MR imaging—including MRCP—may be performed alone or in combination for a more comprehensive diagnostic assessment of complications of hepatic hydatid cysts. In addition, an invasive combined diagnostic and therapeutic approach is usually indicated in specific cases including ERCP in case of suspicion of communication with biliary tree and surgery for peritoneal dissemination.

## Conclusions

Complications of hepatic echinococcosis occur in about one third of patients and may potentially be life-threatening if not promptly diagnosed. Clinical diagnosis of complicated hepatic hydatid cysts may be difficult because of non-specific symptoms. Adequate knowledge of diagnostic imaging clues of complications of hepatic echinococcosis is crucial to guide the clinical, radiological, and therapeutic management.

## Data Availability

Data sharing is not applicable to this article as no datasets were generated or analyzed during the current study.
